# Human iPSC-Derived Glia as a Tool for Neuropsychiatric Research and Drug Development

**DOI:** 10.3390/ijms221910254

**Published:** 2021-09-23

**Authors:** Johanna Heider, Sabrina Vogel, Hansjürgen Volkmer, Ricarda Breitmeyer

**Affiliations:** Molecular Neurobiology, NMI Natural and Medical Sciences Institute at the University of Tübingen, 72770 Reutlingen, Germany; johanna.heider@nmi.de (J.H.); sabrina.vogel@nmi.de (S.V.); volkmer@nmi.de (H.V.)

**Keywords:** iPSC, neuropsychiatric diseases, schizophrenia, autism spectrum disorder, inflammation, CNS, microglia, astrocytes

## Abstract

Neuropsychiatric disorders such as schizophrenia or autism spectrum disorder represent a leading and growing burden on worldwide mental health. Fundamental lack in understanding the underlying pathobiology compromises efficient drug development despite the immense medical need. So far, antipsychotic drugs reduce symptom severity and enhance quality of life, but there is no cure available. On the molecular level, schizophrenia and autism spectrum disorders correlate with compromised neuronal phenotypes. There is increasing evidence that aberrant neuroinflammatory responses of glial cells account for synaptic pathologies through deregulated communication and reciprocal modulation. Consequently, microglia and astrocytes emerge as central targets for anti-inflammatory treatment to preserve organization and homeostasis of the central nervous system. Studying the impact of neuroinflammation in the context of neuropsychiatric disorders is, however, limited by the lack of relevant human cellular test systems that are able to represent the dynamic cellular processes and molecular changes observed in human tissue. Today, patient-derived induced pluripotent stem cells offer the opportunity to study neuroinflammatory mechanisms in vitro that comprise the genetic background of affected patients. In this review, we summarize the major findings of iPSC-based microglia and astrocyte research in the context of neuropsychiatric diseases and highlight the benefit of 2D and 3D co-culture models for the generation of efficient in vitro models for target screening and drug development.

## 1. Introduction

Studying neurobiological and neuroimmunological processes of neuropsychiatric disorders is limited by a lack of appropriate experimental models. For many years, the study of neuropsychiatric diseases was restricted to the use of human post-mortem brain tissue or animal models. Even though these studies have provided us with valuable insights into the underlying pathology of these diseases, the significance is limited. Post-mortem studies only provide evidence for disease end-stage pathology, but not for pathological mechanisms at earlier time points during disease progression. Animal models are informative for dynamic disease processes, but are limited in translation and comparability to the human brain, ultimately, with the consequence that the field suffers from a high failure rate in drug development. Recent development and progress in stem cell technologies, such as the availability of human induced pluripotent stem cells (iPSC) and numerous differentiation protocols, now fosters the hope for more relevant in vitro test systems [1]. Most importantly, human iPSC offers a novel model system with the possibility to study human developmental processes in the context of schizophrenia (SCZ) and autism spectrum disorder (ASD) in vitro.

iPS cells can be differentiated into any cell type, allowing researchers to model a variety of different human pathologies by the analysis of disease- and patient-specific key cell types in vitro. Patient-derived iPSC are especially useful to model idiopathic or complex polygenic diseases, since they retain the individual genetic background of the donor [2]. Therefore, iPSC-based models represent a tool to understand the individual genetic impact on pathogenesis and offer the opportunity to develop personalized treatment options. Here, we review recent data on the use of human iPSC to model schizophrenia and autism spectrum disorder. Under the premise that inflammatory mechanisms contribute to impaired synaptic function, which has been observed in SCZ and ASD patients, the current knowledge on the specific roles of microglia and astrocytes as the main drivers of inflammation in the central nervous system (CNS) will be discussed.

### Clinical Observations and Synaptic Pathologies of Neuropsychiatric Disorders

SCZ and ASD belong to a highly heterogeneous group of neurodevelopment disorders that are characterized by cognitive and behavioral abnormalities and are caused by a complex interplay between various genetic and environmental risk factors. Both diseases are of high prevalence (~ 1% for SCZ, ~ 2% for ASD) and currently there is no cure available for any of the diseases [3,4]. SCZ and ASD are signified by specific symptomatic overlaps, including social withdrawal, anxiety and cognitive impairments, while positive symptoms can vary in patients [5,6,7,8]. SCZ patients can experience positive symptoms like hallucinations and delusions in addition to social withdrawal, flattened affect and lethargy, which are clinical features described as negative symptoms. Lastly, cognitive symptoms include impaired working memory, attention, visual and verbal learning, or logical reasoning [5,9]. The onset of symptoms typically occurs in late adolescence or early adulthood, and the prevalence is similar for both sexes [5,10]. In ASD, negative symptoms include impaired social and communication skills similar to SCZ, while ASD patients do not experience hallucinations or delusions. In ASD, positive symptoms encompass restricted, repetitive or stereotyped behavior regarding speech, gestures, or facial expression, and also in interests and activities. In most cases, the disease manifests early during childhood, with symptoms occurring from as early as 12 months on throughout the patients’ lifetime [11]. The prevalence of ASD is three to four times higher in males than in females [12].

Even though patients with SCZ and ASD show a diverging symptomatic picture, both diseases share altered neuronal connectivity and network activity as two of the major neuronal pathologies. In SCZ, spine densities were found to be reduced, especially in layer 3 of the neocortex, presumably due to the altered formation and pruning of dendritic spines during neurodevelopment [13]. Likewise, an impairment of different types of GABAergic interneurons is suggested to contribute to synaptic deficits that may account for impaired gamma oscillatory activity related to working memory deficits in SCZ [14]. Besides a suggested contribution to disturbed neurogenesis and neurite outgrowth, increased spine densities were observed in the cerebral cortex of ASD patients [15].

In iPSC-derived neurons, synaptic phenotypes of SCZ and ASD have been successfully reproduced. Decreased neurogenesis, reduced neurite outgrowth, and decreased levels of the post-synaptic protein PSD-95 as a surrogate for improper network development have been reported for both diseases [16,17,18]. iPSC-derived neuronal progenitor cells (NPCs) generated from ASD patients displayed increased proliferation and a reduction in excitatory NPC markers. Moreover, ASD-NPCs showed GABAergic differentiation deficits and reduced numbers of excitatory presynapses when differentiated into neurons [19].

While neuronal phenotypes in SCZ and ASD diseases have been studied extensively, increasing evidence has linked deregulation of the immune system to neuropsychiatric disorders [20]. Glial cells, such as microglia and astrocytes, represent the innate immune cells of the CNS and closely interact with neurons and their synapses, contributing to proper synaptic connectivity and neuronal activity during development and maturation [21]. Neuroinflammation is typically a precisely regulated and balanced process, mainly mediated by microglia and astrocytes, which play essential roles in tissue homeostasis and neuroprotection. Detrimental effects of uncontrolled inflammation are suggested to contribute to the onset and development of several neuropsychiatric or neurodegenerative diseases [22]. For SCZ and ASD, it is hypothesized that microglial activation in combination with additional neuroinflammatory mechanisms affect neuronal integrity and synaptic plasticity by aberrant cytokine production complements protein release and synaptic pruning. It is further suggested that the pathological loss of prefrontal cortical volume and decreased synaptic density found in patients diagnosed with SCZ is connected to increased microglial density and activation [23,24]. To further understand and evaluate the inflammatory state of glial cells in individual neurodevelopmental diseases, iPSC-derived glia represent an important human in vitro approach for biomarker discovery and drug development. Therefore, studying the mutual interaction between microglia, astrocytes and neuronal cells may contribute to an improved understanding of neuropsychiatric disease onset, progression, and symptom severity.

## 2. Microglia in Health and Disease

### 2.1. Functions of Microglia in the Developing and Adult Brain

Over the past decades, rodent models, post-mortem tissue analysis, and in vivo neuroimaging studies have increased the understanding of aberrant neuron-microglia crosstalk and microglia-mediated neuroinflammation in the context of SCZ or ASD. Microglia are the resident, innate immune cells of the human brain, and their main functions comprise maintaining function and homeostasis within the developing, the maturing and the inflamed brain. Primarily, microglia act as the immunological and phagocytic defense against bacterial or viral agents in the developing and adult CNS. Rodent studies identified microglial development from mesodermal, primitive myeloid precursor cells within the yolk sac [25]. At embryonic day 8.5 in mice, primitive microglia migrate into the developing CNS before the blood-brain-barrier forms [26,27,28], a pathway that is conserved in human embryonal development [29]. In the brain, microglia are able to self-renew from local progenitor pools and are independent from the infiltration of circulating monocytes [30,31]. Therefore, microglia are the only non-neuronal cell type in the human CNS. Transcriptome analysis identified diverse sub-phenotypes of microglia in the CNS that change over time, highlighting their dynamic role not only during inflammation, but also during CNS development and maturation [32,33].

Microglia are highly mobile and constantly survey their environment with the ability to quickly adapt to local changes [34]. As immune cells, microglia are able to respond to local inflammation and infection by morphological changes and the release of inflammatory agents to prevent neurotoxicity or neurodegeneration. Similar to peripheral macrophages, microglia become activated through specific pattern recognition receptors (PRR) to release inflammatory cytokines and chemokines [35]. PRRs include Toll-like receptors (TLR) that are essential for sensing and responding to bacterial (TLR4) and viral (TLR3, TLR9) pathogens [36]. Microglial activation includes a change in morphology, increased reactive oxygen production, enhanced cytokine release, complement proteins, and phagocytosis of pathogens or cellular debris [37].

Besides being the major player during CNS immune defense, microglia play a central role in early neurodevelopment, mainly by sculpting the immature neurocircuitry [38]. The refinement of synaptic connections between neurons, dendrites, and synaptic terminals is called synaptic pruning, and it describes a necessary process for the proper elimination of excessive and inactive synapses, while active synapses are strengthened [38,39,40]. Synaptic pruning is a phagocytic mechanism to label synapses, apoptotic cells, or other cellular structures for degradation by microglia. CNS synapse elimination has been found to be governed by the complement system and its so called “eat-me” molecules C1q, C3, or C4 that mark synapses for elimination by microglia during neurodevelopment and aging [41,42]. Accordingly, mouse models proved that inhibition of C1q or C3 reduced microglia-mediated synapse loss and revealed aberrant synaptic elimination e.g., in a mouse model for Alzheimer’s Disease [43].

### 2.2. Microglia in Schizophrenia 

Several lines of evidence suggest an increased inflamed state in SCZ patients. RNA sequencing of peripheral blood-monocyte-derived microglia-like cells from twenty SCZ patients revealed differential expression of inflammatory genes such as CCR2, CD44, CD95, or HLA-DR compared to control cells. Similarly, patient-derived microglia showed an increased baseline expression of pro-inflammatory cytokines like IL-1β, IL-6, and TNFα and an increased response to LPS [44]. Additional studies analyzing cerebrospinal fluid (CSF) samples from SCZ patients reported increased levels of albumin and enhanced release of the pro-inflammatory cytokines IL-6, IL-8, and TNFα [45,46], leading to an impairment of the blood-brain-barrier and interfering with its function to isolate the CNS from harmful immunological responses [47,48].

Further evidence for a role of neuroinflammation in SCZ was provided by genome-wide association studies that identified a single nucleotide polymorphism within the major histocompatibility complex (MHC) of SCZ patients, a region that harbors genes with the strongest risk association [49]. An increased expression of *C4* that is located within the MHC locus is suggested to contribute to enhanced SCZ susceptibility [50]. Additionally, other clinical findings revealed increased C4 levels in the CSF of SCZ patients [51]. Altogether, it is suggested that a deregulated expression of proteins of the complement system, which is responsible for marking synapses for degradation by activated microglia, contributes to aberrant synaptic elimination in schizophrenia.

The involvement of microglia in aberrant synapse elimination motivated the application of anti-inflammatory drugs for SCZ. Minocycline is a semisynthetic tetracycline that is able to pass through the blood-brain barrier and has gained attention as a potential new agent to treat neuroinflammatory mechanisms in neurodegenerative and neuropsychiatric disorders [52]. Significant reductions in negative symptom severity have been observed in patients with schizophrenia along with a strong neuroprotective effect of minocycline when applied as adjunctive therapy [53,54]. Currently, anti-inflammatory drugs and agents such as minocycline are of high interest for clinical trials targeting neuroinflammatory processes in SCZ mediated by reactive microglia [55].

### 2.3. Microglia-Based Models for Schizophrenia 

Since brain biopsies have so far represented the only source for the derivation of primary human microglia, several efforts have been made to overcome the limitations of available human microglia for in vitro studies. Immortalized microglial cell lines were applied for initial experiments, however, their transcriptomic signatures revealed the absence of key microglia gene expression, underlining their immaturity [56,57,58]. Alternatively, microglia-like cells matured from blood-monocytes may represent another possibility to analyze molecular and pathological phenotypes in patient-derived cells [44,59]. Given the divergent ontogeny of microglia in comparison to peripheral macrophages, the use of blood monocyte-derived microglia-like cells is, however, limited [60].

To get access to a human cellular system, iPSC-derived models of differentiated microglia offer a powerful tool to understand the biology of microglial action and dysfunction. Over the past years, several protocols for the generation of functional microglia from iPSC have been published, differing significantly in the yield, homogeneity, and maturity of the final microglia population [61,62,63,64,65,66]. A detailed discussion of the advantages and disadvantages of individual differentiation protocols were comprehensively discussed elsewhere [67,68]. However, it is a prerequisite that protocols for the generation of functional, mature microglia are robust and reproducible across laboratories and that the pool of generated microglia share the same transcriptomic signature found in in vivo microglia. The majority of protocols yield microglia that are similar to tissue-resident macrophages by sequential exposure to specific growth factors and cytokines with a final maturation in media containing IL-34, TGFβ1, and macrophage colony stimulating factor. RNA sequencing revealed an immature status similar to fetal microglia, whereas a co-culture of microglia with neurons resulted in a more mature phenotype that was closer to brain-resident microglia [62]. Thus, studying microglia requires interaction with other CNS cell types like neurons, astrocytes, and oligodendrocytes, to achieve a suitable phenotype.

Application of iPSC-based models for microglia-neuron interactions in SCZ revealed increased synaptic pruning by microglia derived from SCZ patients [59,69]. The authors have made use of blood-derived monocytes matured into microglia-like cells and subsequently co-cultured with iPSC-derived cortical excitatory neurons. Synapse elimination and enhanced PSD-95 uptake by SCZ microglia-like cells was highly increased as compared to control cells. Most interestingly, application of the anti-inflammatory drug minocycline reversed excessive synaptic elimination in a dose-dependent manner in accordance with findings previously observed in patients [59]. Therefore, microglia-neuron co-culture models appear to be a valid tool for anti-inflammatory drug testing.

**Figure 1:**
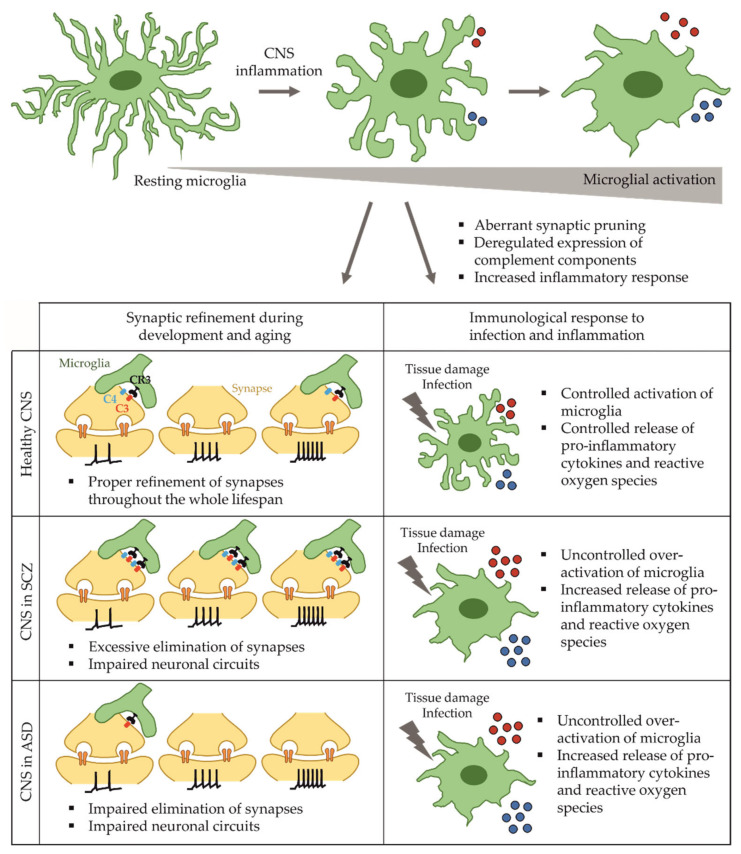
Hypothetical shared and differing pathological phenotypes of microglia in SCZ and ASD. Synaptic pruning of inactive, weak or excessively active synapses is a necessary process during neurodevelopment and aging. For SCZ, it is suggested that overactivated microglia degrade too many active and necessary synapses in a complement-dependent mechanism, thereby impairing neuronal circuits and function. In ASD patients, synapse numbers were found to be increased, indicating reduced synapse elimination by microglia. In both disorders, microglia respond to pro-inflammatory stimulation with an increased immune response accompanied by an enhanced release of pro-inflammatory cytokines and reactive oxygen species, ultimately inducing an impairment of the blood-brain-barrier and the whole CNS.

In summary, SCZ is associated with mutations in several immune-related risk genes and an increased inflammatory state, which are represented in patient-derived iPSC. Given the prominent role in synaptic pruning, a link between the pro-inflammatory response of activated microglia and synapse elimination in SCZ has been provided in microglia matured from iPSC (see Figure 1). Faithful recapitulation of inflammatory phenotypes in SCZ patient-derived, iPSC-based culture systems motivates the use of such systems for the development of anti-inflammatory drugs.

### 2.4. Microglia Dysfunction in Autism Spectrum Disorder 

The involvement of microglia-mediated neuroinflammation in the pathology of ASD was shown by an increased activation of microglia and increased microglial cell density in dorsolateral prefrontal cortex of the human brain [70,71]. In contrast to SCZ findings, human post-mortem studies and animal models revealed enhanced dendritic spine density along with impaired synaptic pruning in layer V neurons of the temporal lobe in ASD patients [15]. Transcriptome analysis revealed a deregulation of genes involved in synaptic function as well as an upregulation of microglial genes as further evidence for a general inflammatory state in ASD [72,73]. As described above for SCZ patients, single nucleotide polymorphisms within the MHC locus were also detected in ASD patients with a deregulated expression of complement component C4 [74,75], indicating that synaptic pruning may also be affected in ASD. Deregulated levels of pro-inflammatory cytokines were detected in the blood of ASD patients, further strengthening the suggestion of increased inflammation in ASD [76]. Likewise, DNA methylation studies revealed a deregulation of several genes involved in microglial specification and synaptic pruning. Microglial activation was significantly increased, while the amount of cells did not change [77]. Microglia are hypothesized to contribute to aberrant synaptic pruning in a complement-dependent manner (see Figure 1), but results derived from relevant human tissue are missing and iPSC-based models using microglia to study ASD have not been established yet.

Similar to SCZ, a deregulated microglial complement system, especially regarding component C4, might have an impact on synaptic pruning in ASD. However, further work is required to understand how synaptic pruning by deregulated microglia contributes to the contrasting effects on dendritic spines that results in increased or decreased spine density in ASD or SCZ patients, respectively. Anti-inflammatory interference with aberrant synaptic pruning in the progression of SCZ and ASD may have beneficial effects on symptom progression and severity, and holds great promise for personalized therapy in high-risk individuals and drug development.

## 3. Astrocytes in Health and Disease

### 3.1. Functions of Astrocytes in the Developing and Adult Brain

Astrocytes represent the most abundant cells in the human CNS known to be involved in various biological processes [78]. During human brain development, the process of neurogenesis precedes the generation of glial cells [79]. Astrocyte precursor cells originate from either NPCs or radial glia in the ventricular zone of the brain, with astrocyte proliferation peaking within the first postnatal weeks in the rat hippocampus [80,81,82]. Newly generated astrocytes migrate towards the cortex along the processes of radial glial cells to proliferate and mature once they reach their final destination [81]. Functionally, astroglial cells are crucial for the development of maturing neuronal networks. Astrocytes promote synaptogenesis by releasing various molecules, such as brain-derived neurotrophic factor, hevin, thrombospondin, and TGF-β [83]. Hevin, for example, stabilizes the connection of the two trans-synaptic adhesion molecules neurexin and neuroligin [84]. Moreover, astrocytes contribute to the stabilization of AMPA receptors at the post-synapse, thereby promoting synaptic maturation [83]. Further developmental processes in which astrocytes are involved include dendritic spine formation and maturation, neuronal survival and the integration of adult-born neurons into the preexisting neuronal circuitry [85].

In the adult brain, astrocytes continue to play a pivotal role in regulating the brain metabolism and neuronal activity. Astrocytic endfeet are part of the blood-brain-barrier where they mediate cerebral blood flow in response to changes in neuronal activity [86]. Moreover, astrocytes are involved in the regulation of cerebral water and potassium homeostasis [87] and are able to provide lactate to neurons in the event of hypoglycemia [88]. The involvement of astrocytes in synaptic processes has been extensively studied and their contribution to synapse function, formation and elimination, the refinement of neuronal circuits, and neuronal plasticity have been demonstrated in numerous studies [89,90,91,92]. Furthermore, astrocytes express different transporters to take up GABA and glutamate from the synaptic cleft. Intracellularly, these neurotransmitters are broken down into glutamine and subsequently, glutamine is supplied back to neurons [93].

### 3.2. Astrocytes in Schizophrenia

More recently, the role of astrocytes in the pathology of SCZ has gained increasing attention, as evidence is accumulating from genetic and post-mortem studies indicating profound changes in astrocyte gene expression, morphology, and function, which correlate with SCZ. Deregulated expression of a number of astrocyte-related genes including excitatory amino acid transporter 2 (*EAAT2*) [94], d-amino acid oxidase (*DAO*) [95], thrombospondin 1 (*THBS1*) [96], and S100 calcium-binding protein B (*S100β*) [97] were found to be associated with SCZ. Additionally, several post-mortem studies reported alterations of astrocytic protein expression in SCZ patients. Protein levels of two common markers of astrocytes in the CNS, the glial fibrillary acidic protein (GFAP) and the aldehyde dehydrogenase 1 family member L1 (ALDH1L1), were found to be deregulated in different studies in cortical as well as subcortical regions [98,99,100].

Nowadays, it is widely accepted that neuroinflammation is involved in the pathogenesis of SCZ [101]. A model of glial dysfunction in SCZ proposed that the activation of microglial cells by inflammation during development could disrupt the proliferation of glial progenitor cells. This in turn could lead to an altered and/or delayed maturation of astrocytes [102]. In line with this model, there are multiple post-mortem studies showing that the number of astrocytes is altered in a brain-region dependent manner in SCZ. Astrocyte numbers are reportedly reduced in the nucleus accumbens [103], basal nuclei [104], cingulate cortex [105], and the substantia nigra [106]. However, an increase of astrocytes was found in the periventricular space [107].

A further possible consequence of CNS inflammation is astrogliosis, which describes a reactive state of astrocytes. Changes in gene expression patterns, as described above, as well as morphological changes and increased astrocyte numbers are characteristics of astrogliosis [108]. In one study, reactive astrogliosis was found in approximately 70% of patients with SCZ in the periventricular zone, basal forebrain, and periaqueductal region [109]. An electron microscopy study reported an increase in astrocyte density and volume in the hippocampus of SCZ subjects compared to control, indicative of local astrogliosis [110]. These results are contrary to the decrease of astrocyte numbers in specific brain areas described above, suggesting a brain-region dependent phenotype.

Overall, there are several lines of evidence indicating that the astrocyte transcriptome, proteome, morphology and, as a likely consequence, the astrocyte function itself, is affected in SCZ. The inflamed state in SCZ patients is suggested to lead to altered glial progenitor proliferation and differentiation deficits, as well as astrogliosis.

### 3.3. Astrocyte-Based Models for Schizophrenia

Astrocytes differentiated from human iPSC provide a valuable tool to study astrocyte function in neuropsychiatric diseases. Importantly, astrocytes enriched from human brain tissue differ significantly from e.g., mouse astrocytes, as they are morphologically more complex and show differences in their transcriptomes and responses to glutamate in vitro [92]. Therefore, using iPSC-derived astrocytes provides an opportunity to create in vitro model systems, providing a better translation to the human brain.

To date, different protocols describing the differentiation of iPSC into astrocytes have been published with varying results in yield and maturity. The first published protocols would take as long as 180 days to differentiate iPSC into immature astrocytes [111]. More advanced protocols were able to overcome this drawback and reduce differentiation time to as little as 30 days by using NPCs as a starting point [112,113]. Detailed descriptions of differences and commonalities between published astrocyte differentiation protocols are described elsewhere [113,114]. Before the start of glial differentiation, iPSC must pass a neuronal progenitor phase. For neural induction, media are usually supplemented with N2, B27, and basic fibroblast growth factor (bFGF). Ciliary neurotrophic factor is then commonly supplied to the medium to direct the cells towards an astrocytic fate [112,113,115,116,117]. In order to validate the successful conversion of iPSC into mature astrocytes, immunocytochemical staining of the mature astrocyte markers GFAP and S100β, transcriptome analysis, and functional assays using calcium imaging are routinely performed. The purity of the differentiated cultures can reach > 90% GFAP/S100β double-positive astrocytes [117].

Studies using human iPSC-derived astrocytes were able to reproduce some of the phenotypes which were observed in post-mortem studies and add to our understanding of the underlying molecular mechanisms. Supporting the notion that astrocytes show differentiation deficits in SCZ, iPSC-derived glial progenitor cells (GPC) from patients with childhood-onset SCZ revealed differentiation deficits both in vitro and in vivo [118]. In vitro, SCZ-GPCs aberrantly expressed genes associated with glial differentiation and synapses. Upon transplantation into myelin-deficient mice, the animals experienced hypomyelination due to the premature migration of GPCs into the gray matter. Moreover, the fraction of engrafted SCZ-GPCs which were GFAP+ and showed typical astrocytic morphology, was lower in SCZ-GPC chimeric mice compared to control, indicating a potential delay in astrocyte maturation in these mice [118]. In a follow-up study, the deregulation of intracellular signaling pathways were shown to contribute to the observed differentiation deficits. iPSC-derived SCZ-GPCs were found to upregulate several transcripts of the bone-morphogenic protein (BMP)-signaling pathway. By knocking down SMAD4, a molecule involved in BMP signaling, the differentiation of SCZ-GPCs was normalized, increasing the fraction of GFAP+ astrocytes derived from SCZ-GPCs [119]. Contrary to these observations, another iPSC-based study reported increased astrocytic differentiation of cells derived from SCZ patients carrying a 22q11.2 microdeletion [98]. Increased astrocyte differentiation was further reported in a second study, in which neurospheres were generated from iPSC derived from SCZ patients and healthy controls. The neurospheres were differentiated into neurons and glial cells, with SCZ-derived neurospheres generating an increased number of astrocytes and a decreased number of neurons. This finding was linked to increased levels of the kinase p38A in the SCZ-neurospheres, which are implicated in the neuron–glia switch during development [98]. Overall, both post-mortem studies and iPSC-based studies report either increased or decreased glial differentiation, which suggests that there might be several different mechanisms underlying these developmental phenotypes.

Most recently, the first drug study using iPSC-derived astrocytes from SCZ patients was published [120]. Several genes related to immune processes were upregulated in SCZ astrocytes, highlighting the inflammatory phenotype associated with the disease [101]. Moreover, intracellular glutamate and D-serine levels were found to be significantly reduced in SCZ-astrocytes compared to control. Interestingly, when SCZ-astrocytes were exposed to the antipsychotic drug clozapine, D-serine levels were normalized in astrocytes derived from clozapine-responders, but not in clozapine non-responding SCZ patients [120]. This study demonstrates the usability of iPSC models to study mechanisms of drug response.

Besides patient-derived models, isogenic SCZ disease models have been employed to assess astrocyte function. Most isogenic diseases models focus on the SCZ risk gene *DISC1*, which is implicated in a variety of functions, including neuronal migration, differentiation, and neurite outgrowth [121]. More recently, research started to focus on the role of DISC1 in astrocytes as well. The primary astrocytes of *DISC1* mutant mice show reduced glucose uptake and lactate production, which affects their metabolic support of neurons [122]. In co-cultures of healthy primary neurons and *DISC1* mutant astrocytes, less excitatory synapses and less dendritic complexity were reported [123]. Currently, there is still a lack of iPSC-based studies investigating the effect of *DISC1* mutations in astrocytes, a topic which should be addressed by future research. However, one study reported elevated lactate levels in cortical neurons differentiated from one SCZ patient carrying a *DISC1* mutation. This result suggests that there might be alterations in astrocytic lactate production and its supply to neurons [124].

*DISC1* is also known to be a risk gene for bipolar disorder (BPD) [125,126], another neuropsychiatric disorder genetically related to SCZ. BPD is a chronic disease characterized by alternating episodes of mania and depression with varying duration [127]. Like SCZ, BPD has been associated with increased inflammation and increased astrocyte reactivity [128]. Gene expression analysis using post-mortem BPD tissues revealed an elevated expression of astrocytic marker genes in cortical and subcortical regions similar to findings of SCZ tissues [100,129]. This finding indicates an increased astrocyte reactivity, possibly provoked by inflammation. An inflammatory state could in turn further stimulate the inflammatory response of astrocytes, leading to alterations of neuronal morphology and activity. This concept was demonstrated with the first iPSC-derived models for BPD, revealing a distinct inflammatory response upon IL-1β stimulation as compared to control astrocytes [130]. BPD-astrocytes significantly reduced the activity of co-cultured control neurons, and pro-inflammatory stimulation exacerbated this effect in an IL-6 dependent manner. Treatment with an anti-IL-6-antibody rescued the phenotype of impaired co-cultured neurons. Overall, this study provides evidence for an increased inflammatory state in BPD which negatively affects neuronal activity via the activation of astrocytes [130].

### 3.4. Astrocyte Dysfunction in Autism Spectrum Disorder 

Similar to SCZ, the activation of astrocytes has been proposed to lead to changes in astrocyte morphology and function in the context of ASD [131]. Post-mortem brain tissue analysis of ASD patients has revealed an increase in GFAP expression in different brain regions indicative of astroglial activation [132,133]. Moreover, GFAP levels are elevated in CSF of ASD patients [134]. Supporting this activated state, several inflammatory cytokines and chemokines were increased in ASD brain tissue across different studies [133,135]. Taken together, it is suggested that activated astrocytes and microglia release inflammatory cytokines and chemokines and thereby affect neuronal integrity, synapse formation and function in patients with ASD [131].

This concept was confirmed using iPSC-derived astrocytes [112]. Astrocytes generated from three patients with non-syndromic autism showed increased levels of the pro-inflammatory cytokine IL-6, reactive oxygen species, and a tendency towards increased glutamate levels. Next, ASD-astrocytes were found to negatively affect the neuronal morphology of co-cultured control and ASD-neurons by reducing dendritic complexity and arborization, while control astrocytes improved the morphology of ASD-neurons. Reduced synapse densities suggest that astrocytes interfere with neuronal synaptogenesis in ASD. Likewise, pure ASD-neurons cultured in absence of astrocytes displayed a reduction of pre- and post-synaptic proteins, underlining the important contribution of astrocytes to synaptogenesis. When control astrocytes were co-cultured with ASD-neurons, the number of synaptic puncta was increased, which was not the case in a co-culture with ASD-astrocytes. IL-6 application led to a decrease in synapses in the control neurons, whereas treatment with anti-IL-6 resulted in an increase in synaptic structures. Therefore, increased IL-6 secretion from ASD-astrocytes seems to contribute to ASD-related neuronal phenotypes by a reduction of synaptic numbers [112]. The importance of the innate immune system on brain development was most recently confirmed by a study analyzing complement protein expression in iPSC-astrocytes from ASD patients. Complement component C4 expression was significantly decreased in ASD-astrocytes in comparison to the control astrocytes [136]. These findings further contribute to a wider understanding of aberrant complement-mediated synaptic loss in ASD.

Rett syndrome (RTT) is a specific neurodevelopmental disorder that is classified as an autism spectrum disorder. RTT is much more rare than ASD or SCZ and is a monogenetic disease caused by loss-of-function mutations in the methyl-CpG binding protein 2 (*MECP2*) gene [137]. In the first years of life, patients with RTT show a loss of previously acquired motor and language skills, and experience breathing abnormalities and seizures [138]. hiPSC-derived *MECP2* mutant astrocytes display severe effects on the development and function of co-cultured murine hippocampal neurons which show decreased neurite lengths, smaller somata, fewer neuronal terminals and reduced activity, a finding similar to the observations made using ASD-astrocytes. Co-culture of neurons with conditioned media from RTT-astrocytes reproduced these findings, highlighting the glial contribution to RTT [139]. Another study showed an increase in astrocyte-specific differentiation in iPSC-derived RTT-neurospheres, replicating previous findings from mouse models [140,141,142].

**Figure 2:**
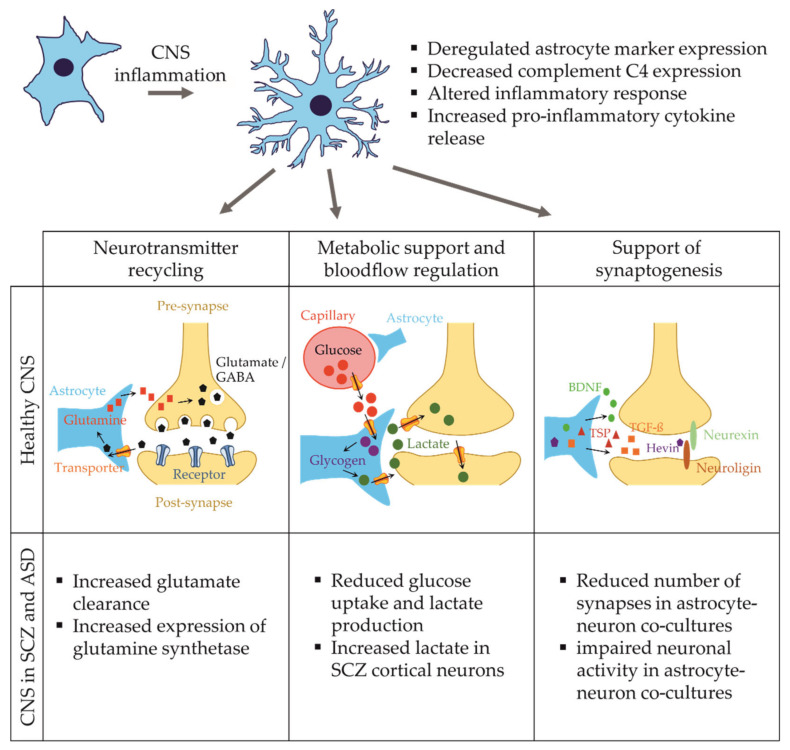
Overview of major astrocyte functions in the healthy brain and in neuropsychiatric disorders. Astrocytes are closely associated with pre- and postsynaptic sites, where they are involved in neurotransmitter clearance and recycling, the metabolic support of neurons, and the formation and stabilization of synapses mediated by the release of various molecules. In neuropsychiatric diseases, astrocytes are activated by inflammation and undergo changes to their morphology and expression profile, showing altered astrocytic differentiation and inflammatory responses. Consequently, these changes affect the functionality of astrocytes, ultimately leading to impairments of neuronal function.

As outlined above, changes in astrocyte morphology and function have been linked to neuropsychiatric diseases using iPSC-models, making astrocytes an attractive target for drug screening. There are different readouts, which can be used to perform drug studies using iPSC-astrocytes. The expression of astrocytic proteins (e.g., GFAP, S100β) and astrocyte morphology are two parameters that are directly affected by inflammation and are simple to assess. Moreover, astrocytic Ca^2+^ signaling or the study of the astrocyte secretome are appropriate readouts to evaluate inflammation-related effects. In two recent studies, iPSC-astrocytes were exposed to the pro-inflammatory molecules TNF-α, IL-1 α, IL-1β, and C1q to induce a pro-inflammatory phenotype of the cells [143,144]. Indeed, the astrocytes responded by morphological, transcriptomic, and proteomic changes characteristic of an acute inflammation (see Figure 2). Such approaches provide useful tools for future drug studies to model inflammation in vitro and investigate the effect of anti-inflammatory compounds in neuropsychiatric diseases.

## 4. Lessons from iPSC Models for Research and Drug Development

While the use of iPSC holds great promise in providing valid human test models in vitro, obvious limitations and challenges prevail. One problem is the relatively immature nature of cell types differentiated in vitro. A further fundamental problem is associated with the use of reductionist in vitro models signified by the presence of only one or few different cell types at best that significantly contrasts with the huge complexity of the human CNS in vivo. It is beyond any doubt that brain functions rely on highly diverse cell types, their individual specific functions and reciprocal communication among them. It is therefore conceivable that enhancing the complexity of human model systems will improve model validity, especially for the study of CNS disorders and concomitant drug evaluation. This can be achieved by introducing different cell types in co-culture systems. Alternatively, the use of three-dimensional brain organoids offers a further opportunity to specifically study individual cellular functions and communication between different cell types in vitro.

### 4.1. Multi-Culture Models to Study Reciprocal Cross-Talk in Diseases of the Central Nervous System 

**Figure 3:**
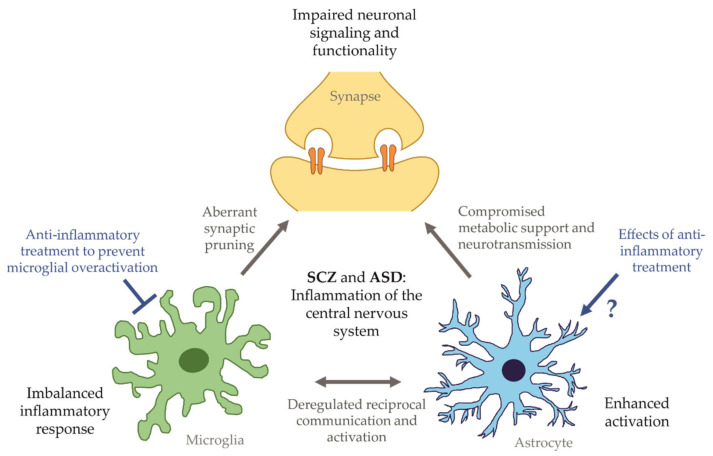
Impaired communication of glia cells and neurons as a consequence of CNS inflammation. The imbalanced reciprocal activation of microglia and astrocytes may lead to the development of neuronal pathologies, contributing to the onset and progression of neuropsychiatric disorders like SCZ and ASD.

As an approach to study cell–cell interactions, co-culture systems are beneficial for the differentiation of mature and functional microglia, as outlined above in the example of interactions between microglia and neurons [62]. However, the precise nature of such interactions is only partially understood. Besides interactions of components of the complement system, the secretion of soluble growth factors, cytokines, metabolic mediators, or extracellular vesicles primarily governs the communication between neurons, astrocytes, and microglia. Exemplarily, proteome analyses revealed that extracellular vesicles released by microglia propagate inflammatory reactions onto astrocytes after ATP-stimulation [145]. Approaches for further expansion to three cell types including neurons, astrocytes, and microglial cells have been introduced. To this end, separate differentiations of astrocytes, microglia, and neurons are necessary to induce the individual phenotypes in vitro, but subsequent seeding into co-cultures enables researchers to perform highly controlled experiments with precise cellular ratios. A first iPSC-based tri-culture model consisting of microglia, astrocytes, and neurons at a ratio of 2:1:8 displayed increased pro-inflammatory cytokine release and secretion of the complement component C3 after LPS treatment [146]. C3 levels were only detectable in cultures containing microglia, and tri-culture setups even potentiated C3 levels significantly. In combination with a C3 knockout iPS cell line, it was shown that astrocytic and microglial C3 contribute to overall C3 potentiation, demonstrating reciprocal signaling and activation between astrocytes and microglia. Additionally, astrocyte-conditioned medium increased C3 levels in microglia, indicating that astrocyte-secreted soluble molecules are able to separately activate microglia [146]. These initial findings highlight the power of iPSC-derived multi-cellular culture systems that offer the opportunity to identify specific individual or synergistic cellular mechanisms and to discriminate pathological phenotypes in mono-, co- or tri-culture models. Especially in the context of neuropsychiatric disorders, patient-derived iPSC models can increase our understanding of the effect of irregularly activated glia cells on neuronal function and synaptic pruning (see Figure 3).

### 4.2. Opportunities and Limitations of iPSC-Based Models for Drug Development 

There is much interest in defining cellular phenotypes relevant in the context of individual mental health disorders to create an in vitro system with functional readouts for high-throughput drug testing. For the assessment of impaired neuronal phenotypes, several assays like neurite outgrowth or the determination of synapse densities are available to study inter-patient or inter-disease differences. Additionally, neuronal functionality was determined by electrophysiological recordings or calcium imaging to address neuronal deficits and impairments in different disease states [16,17,147,148]. When studying non-neuronal pathologies in CNS disorders, microglia and astrocytes can be employed to analyze activation patterns, inflammatory responses and aberrant signaling. Initial immunological assays revealed aberrant synapse elimination by SCZ-microglia, which could be reversed by anti-inflammatory treatment with the antibiotic minocycline [59]. Multi-omics platforms comprising metabolomic, transcriptomic, genomic, or epigenomics studies offer diverse approaches to determine disease-related phenotypes. Single cell RNA sequencing and optogenetic or chemogenetic toolkits allow for the functional characterization of heterogeneous cells.

Among all these possibilities and available tools, it is probably the hardest challenge to identify the most suitable assay with a distinct disease-relevant phenotype. There is great interest in using disease-specific phenotypes for high-throughput compound screening to identify novel drug targets [149]. The determination of highly precise and distinct pathological phenotypes of schizophrenia or autism spectrum disorders using iPSC-models is limited by the heterogeneous basis of mental health disorders among patients and variations among different iPS cell lines derived from the same donor. However, given the huge genetic variability among different neuropsychiatric patients, it requires large cohorts of patients and respective control lines to detect common deregulated target mechanisms. This process is highly costly and time consuming. Therefore, the use of a few individually mutated isogenic cell lines is often preferred for diseases signified by mutations of high penetrance [150]. These cell lines are usually derived from healthy control donors and single mutations are inserted into specific disease-associated genetic loci, while the genetic background remains conserved.

Despite all its promises and the sophisticated analyses available, disease modeling based on patient-derived or isogenic mutated iPS cell lines requires further improvement. Highly standardized and robust cell systems need to be generated to establish reliable and reproducible test systems that can routinely be employed across laboratories for disease modeling and ultimately serve for high-throughput drug screening.

### 4.3. Brain Organoids to Study 3D Neuron-Glia Interactions In Vitro

A further degree of complexity has been achieved with the development of 3D brain organoids. About a decade ago, scientists began to generate iPSC-based structural counterparts of the brain in vivo to study cell interactions in a tissue-relevant and organ-like structure, so-called brain organoids [151,152,153,154,155,156]. This technology allows for the study of early development of the human brain [153]. While 2D cultures rely on the random interaction of applied cell types, brain organoids display organized structures representing the layering of the brain. This emerging multicellular system offers the possibility to model dynamic processes and changes in neuropsychiatric and neurodegenerative diseases in vitro.

Two basic types of protocols are now available to generate brain organoids. The unguided method is based on the spontaneous differentiation of iPSC to form self-patterned structures, whereas the guided method needs external patterning factors applied as a cocktail to developing organoids in order to induce differentiation into specific lineages of the neuroectoderm. The mixture of small molecules, growth factors and cytokines largely differs across differentiation protocols. Pioneering work generated heterogeneous brain-like tissues with distinct brain regions like forebrain, midbrain, and hindbrain as well as specific subregions such as the various cortical lobes, the choroid plexus, and the retina [153].

For the study of glia-neuron interactions in 3D, several efforts have been made to establish culture conditions suitable to bring all cell types of interest together. Astrocytes and neurons develop from a common neuroectodermal origin and via a neuronal progenitor state. In 3D cortical spheroids, non-reactive astrocyte-like cells were found to rise in number after 50 days and to promote synapse formation and amplify calcium signaling in neuronal networks [155]. The differentiation of astrocytes in long-term cultured human cortical organoids closely resembles the development of human fetal astrocytes, as they evolve from a mainly fetal to a more and more mature astrocyte-like state. These findings indicate that organoid-derived astrocytes represent a valuable approach for studying neurodevelopmental processes and disease modelling [157].

The challenge of co-differentiating microglia in brain organoids lies within the different origins of neurons and microglia. Whereas guided cerebral organoid differentiation mainly yields cells from the neuroectodermal lineage, microglia originate from the mesodermal lineage. Therefore, a commonly used approach is to differentiate brain organoids and microglia separately and to seed them into co-culture at a given time point to study neuron-glia interactions. Microglia are able to infiltrate into the neuronal environment and retain functions similar to their in vivo counterparts like the phagocytosis of dead cells and debris and synaptic refinement for neuronal circuit maturation [66,158]. In contrast to a separate pre-differentiation and subsequent seeding into co-culture, a cerebral organoid protocol was published that describes the innate development of microglia by day 52 in vitro through the reduction of specific neuroectoderm-inducing stimulants [159]. Intrinsically developed microglia formed functional connections to neuronal networks by the phagocytic uptake of PSD95 positive structures, and response to LPS stimulation by increased cytokine release. This undirected model is of special interest to study aberrant synaptic pruning in the context of neurodevelopment disorders such as SCZ and ASD [159].

### 4.4. Brain Organoids for Neuropsychiatric Research and Drug Development 

CNS organoids provide a promising approach for drug testing. As a first proof-of-concept, forebrain organoids were transiently exposed to different strains of Zika virus to study early deficits in prenatal brain development. Viral infection resulted in decreased organoid size, increased cell death and significantly reduced neuronal layer thickness [156].

As a neurodevelopmental disorder, SCZ is associated with aberrant brain development [160]. Cerebral organoids from SCZ patients revealed an increased circulation and disorganized migration of NPCs throughout all zones of the organoids. Additionally, precursors of cortical neurons were lost within the top cortical layers, while deep subcortical neurons were misplaced. These findings indicate aberrant neurogenesis in organoids derived from SCZ patients [160]. Furthermore, aberrant expression of genes involved in neurogenesis and impaired oxygen consumption were found in cerebral organoids generated from SCZ patients. Electrophysiological recordings revealed no difference in baseline activity upon comparison of SCZ to control organoids, but a decreased response to electrical stimulation was reported [161].

For ASD, telencephalic organoids derived from idiopathic patients revealed an enhanced differentiation into GABAergic neurons next to a consistent differentiation towards glutamatergic neurons. This imbalance of excitatory and inhibitory neurons resulted from severely deregulated expression of FOXG1, a transcriptional factor essential for telencephalic development. Subsequent transcriptome analysis showed significant changes in the transcriptional regulation of cell proliferation, neuronal differentiation and synaptic transmission [162]. A different model for ASD comprises mutations within the risk gene chromatin-investigated factor 8 (*CHD8*). Transcriptome analysis of 50 day old cerebral organoids revealed an implication of the transcriptional regulator CHD8 in the deregulation of several neurogenesis-associated genes, thereby inducing impairments in neuronal differentiation and brain development [163].

While iPSC-derived brain organoids may represent an improved model for neuropsychiatric disorders in vitro, several limitations need to be overcome for feasible drug testing approaches. Transcriptomic comparisons of iPSC-derived organoids and primary human cortical cells further revealed less distinct cellular phenotypes in brain organoids that co-expressed both markers of progenitor cells and mature, differentiated neuronal cells. These findings indicate that iPSC-derived organoids can only represent very early neurodevelopment in vitro [164]. Therefore, long maturation times are necessary to follow and represent the embryonal development of the human brain [155,165]. Another major limitation is the necrotic cores within the center of brain organoids as a result of inefficient nutrient supply to the inner structures from the lack of vascularization.

Further research is needed to establish brain organoid models with higher complexity and improved reproducibility to study neurodevelopmental processes and glia-neuron communication in health and disease. To determine disease-relevant phenotypes regarding neurogenesis, synaptogenesis, or synaptic elimination in different subregions of the brain, functional readouts such as electrophysiological recordings or calcium imaging and immunological assays can be employed. Furthermore, single-cell RNA sequencing and whole-organoid transcriptomics may give rise to understanding specifically deregulated genes in different neurodevelopmental disorders. Additionally, organ-on-a-chip systems in combination with blood-brain-barrier models may hold great potential for neurotoxicity screenings and the discovery of novel therapeutics [166]. It is of note that recent efforts have contributed to the development of automated workflows for human midbrain organoids suitable for high-throughput experimentation in a 96 well format. There is no doubt that drug development will benefit from the further elaboration of such techniques [167].

## 5. Conclusions

iPSC-based models hold great promise for improved cellular in vitro models for SCZ and ASD. In particular, the impact of inflammatory mechanisms conferred by astrocytes and microglia was studied in the context of SCZ and ASD. The two neuropsychiatric diseases share common pathological astrocyte phenotypes, suggesting shared molecular mechanisms linking SCZ and ASD. In contrast, microglial activation and aberrant elimination of synapses is suggested to affect both disorders differently. Overall, an inflammatory state in the brain of patients seems to equally activate astrocytes and microglia, causing abnormalities in individual glia cell marker expression, differentiation and inflammatory responses, and ultimately affecting CNS organization and network functionality.

Clinical findings and initial iPSC-based models have documented the beneficial action of anti-inflammatory treatments, opening now the perspective to identify further promising targets for adjunctive anti-inflammatory therapy of progressing SCZ or ASD. However, it is obvious that the field requires considerable efforts at different levels. Firstly, the basic understanding of the mutual interactions among the contributing cell types is still mostly elusive. Secondly, further studies are required to fine-tune iPSC-derived models to acquire a reproducible and mature system comprising both neuronal and glial cells. Finally, yet importantly, technical hurdles limiting the use of brain organoids such as low reproducibility or insufficient nutrient and oxygen supply, need to be addressed to ultimately improve the fidelity and consistence of iPSC-derived models for neuropsychiatric research and drug development.

## Data Availability

Not applicable.
